# Curcumin Inhibits Transforming Growth Factor β Induced Differentiation of Mouse Lung Fibroblasts to Myofibroblasts

**DOI:** 10.3389/fphar.2016.00419

**Published:** 2016-11-08

**Authors:** Daishun Liu, Ling Gong, Honglan Zhu, Shenglan Pu, Yang Wu, Wei Zhang, Guichuan Huang

**Affiliations:** Department of Respiratory Medicine, Institute of Respiratory Diseases in Zunyi, The First People’s Hospital of Zunyi, The Third Affiliated Hospital of Zunyi Medical CollegeZunyi, China

**Keywords:** curcumin, peroxisome proliferator activated receptor γ, platelet derived growth factor receptor β, pulmonary fibrosis, transforming growth factor β2

## Abstract

Transforming growth factor β (TGF-β) induced differentiation of lung fibroblasts to myofibroblasts is a key event in the pathogenesis of pulmonary fibrosis. This study aimed to evaluate the effect of curcumin on TGF-β induced differentiation of lung fibroblasts to myofibroblasts and explore the underlying mechanism. Mouse lung fibroblasts were cultured and treated with TGF-β2 and curcumin or rosiglitazone. Cell vitality was examined by MTT assay. The secretion of collagen-1 was assessed by ELISA. α smooth muscle actin (α-SMA) was visualized by immunofluorescence technique. The expression of peroxisome proliferator activated receptor γ (PPAR-γ) and platelet derived growth factor R β (PDGFR-β) was detected by PCR and Western blot analysis. We found that curcumin and rosiglitazone inhibited the proliferation and TGF-β induced differentiation of mouse lung fibroblasts. In addition, curcumin and rosiglitazone inhibited collagen-1 secretion and α-SMA expression in mouse lung fibroblasts. Furthermore, curcumin and rosiglitazone upregulated PPAR-γ and downregulated PDGFR-β expression in mouse lung fibroblasts. In conclusion, our study reveals novel mechanism by which curcumin inhibits TGF-β2 driven differentiation of lung fibroblasts to myofibroblasts. Curcumin could potentially be used for effective treatment of pulmonary fibrosis.

## Introduction

Idiopathic pulmonary fibrosis (IPF) is a progressive disease of unknown etiology that can result in respiratory failure (Meltzer and Noble, 2008; Wilson and Wynn, 2009). The principle manifestations of IPF include mild inflammation, fibroblast foci, and extensive extracellular matrix (ECM) deposition in the lung tissue (Pardo and Selman, 2012; Sakai and Tager, 2013). Fibroblasts are major effector cells in the development of pulmonary fibrosis due to their ability to differentiate into myofibroblasts and produce excess ECM components, including collagen and fibronectin (Nathan et al., 2011; Olson and Swigris, 2012; Chełstowska et al., 2014). Myofibroblasts are characterized by the expression of alpha smooth muscle actin (α-SMA), calponin and ECM proteins including Type I and III collagen (Col1A1 and Col3A1), fibronectin and proteoglycan (Nathan et al., 2011).

Transforming growth factor β (TGF-β) is a pleiotropic cytokine that promotes the differentiation of fibroblasts to myofibroblasts and plays a major role in fibrosis. Binding of active TGF-β to its receptor triggers several signaling pathways including Smad pathway and phosphotidylinositol-3-kinase (PI3K)/Akt/Protein Kinase B (PKB) pathway (Guo and Wang, 2008; Assinder et al., 2009; Santibanez et al., 2011). In fetal lung fibroblasts, TGF-β activates Akt signaling via p38 Mitogen Activated Protein Kinase (MAPK) and Focal Adhesion Kinase (FAK; Kulkarni et al., 2011).

Curcumin is derived from the rhizomes of the curcuma longa plant commonly known as turmeric, and has long been used as a medicinal agent in many Asian countries (Maheshwari et al., 2006). Curcumin has shown a variety of potentially beneficial effects, including lower blood pressure, anti-tumor, anti-inflammatory, choleretic, antioxidant, and anti-inflammatory activities (Nakagawa et al., 2014; Seo et al., 2014; Zhang Y. et al., 2014). Notably, curcumin effectively reduces profibrotic effects in fibroblasts *in vitro* via the inhibition of key steps in the TGF-β signaling pathway (Smith et al., 2010). It was reported that the activation of peroxisome proliferator-activated receptor gamma (PPAR-γ) by curcumin blocked platelet derived growth factor (PDGF) signaling pathway in hepatic stellate cells (Lin and Chen, 2008). However, the relationship of PPAR-γ and PDGF signaling pathway is unclear in TGF-β induced differentiation of lung fibroblasts to myofibroblasts.

Here, we report that curcumin exhibited anti-fibrosis effect by inhibiting TGF-β induced differentiation of mouse lung fibroblasts to myofibroblasts and this may be mediated by the regulation of PPAR-γ and PDGFR.

## Materials and Methods

### Cell Culture and Treatment

Mouse lung fibroblast cell line was purchased from American Type Culture Collection (Cat. # CRL-6013^TM^) and maintained in dulbecco’s modified eagle medium (DMEM, Hyclone, USA) supplemented with 10% fetal bovine serum (FBS), 100 U/ml penicillin and 100 μg/ml streptomycin at 37°C in a humidified atmosphere with 5% CO_2_. After the cells reached 70–80% confluence, the cells were digested with 0.25% trypsin to passage, and the medium was changed every other day. Cells were used between passages 5–9. PPAR-γ ligand rosiglitazone (Sigma-Aldrich, USA) was dissolved in DMSO and added to cultured cells to the final concentrations of 5, 10, 20, and 40 μM as described previously (Hogan et al., 2011). Curcumin (Sigma-Aldrich, USA) was prepared in the same manner and added to cultured cells to the final concentrations of 5, 25, and 50 μM as described previously (Hwang et al., 2013). Recombinant mouse TGF-β2 (R&D Systems, USA) was used at a final concentration of 10 ng/ml as described previously (Ma et al., 2014).

### RT-PCR

Total RNA was extracted from mouse lung fibroblasts with Trizol (Invitrogen, USA) following the manufacturer’s instructions and stored at -80°C. The cDNA was synthesized from 2 μl of total RNA and used for PCR using PrimeScript RT-PCR Kit (TaKaRa, Japan). The primer sequences were as follows: PPAR-γ Forward 5′-GCCCTTTACCACAGTTGATTTC-3′ Reverse 5′-GATGCTTTATCCCCACAGACTC-3′; FGF-R1 Forward 5′-GGAGGAGAGAGTGAGAGGATGA-3′ Reverse 5′-TGAGTGGTGTGGGTTTGAATAA-3′; PDGFR-β Forward 5′-GTGGTCCTTACCGTCATCTCTC-3′ Reverse 5′-CTTCTCGCTACTTCTGGCTGTC-3′; β-actin Forward 5′-ATATCGCTGCGCTGGTCGTC-3′ Reverse 5′-AGGATGGCGTGAGGGAGAGC-3′. The parameters for PCR were denaturation at 94°C for 3 min; 30 cycles of denaturation at 94°C for 30 s, annealing at 52°C for 30 s, extension at 72°C for 1 min; final extension at 72°C for 10 min. Aliquots of PCR amplification products were electrophoresed on 2% agarose gels containing ethidium bromide and analyzed by Bio-Rad ChemiDoc image acquisition system and Quantity One (v4.6) software (Bio-Rad, Hercules, CA, USA).

### Western Blot Analysis

Mouse lung fibroblasts were suspended in ice-cold RIPA and lysed in lysis buffer for 30 min. The lysates were centrifuged at 14,000 rpm for 15 min and the supernatants were stored at -80°C. Protein concentrations were determined using the Bradford assay (Pierce, USA). Samples containing 40 μg total protein were separated by SDS-polyacrylamide gel electrophoresis (SDS-PAGE), then transferred to polyvinylidene difluoride membranes (PVDF, Roche) and immunoblotted with anti-PPAR-γ (1:1,000) or anti-β-actin (1:2,000) antibody. The membranes were then incubated with appropriate secondary antibody coupled to horseradish peroxidase and visualized by enhanced chemiluminescence (ECL) detection. β-actin was used as loading control. Densitometric analysis of the bands was performed using Quantity One software.

### Indirect Immunofluorescence Assay

Cells were seeded in 4-well chamber slides and cultured overnight, then treated with rosiglitazone (40 μM) or curcumin (50 μM) for 1 h followed by TGF-β2 (10 ng/ml) treatment for 24 h. Cells were washed, fixed with chilled methanol (-20°C) for 15 min, then washed again and air dried. After equilibration in a humidified chamber, the cells were blocked in 1% BSA in PBS for 30 min, and then stained with anti-α-SMA (Abcam, UK) and anti-mouse AlexaFluor 488 (Invitrogen, USA). Slides were mounted with Prolong Gold supplemented with DAPI (Invitrogen, USA) to visualize the nuclei and analyzed by fluorescence-microscopy using a Leica Microscope.

### MTT Assay

Cells were seeded in 96-well plates and cultured overnight. Next the cells were treated with rosiglitazone (40 μM) or curcumin (50 μM) for 1 h followed by TGF-β2 (10 ng/ml) treatment for 24 h. Viable cells were evaluated with MTT assay kit (Sigma, USA) according to the manufacturer’s instructions. The absorption value was read at 490 nm using a microplate reader.

### ELISA Assay

The supernatants of cell culture were collected and the concentration of collagen I in the supernatants was determined by using Mouse Collagen Type I ELISA Kit (MyBioSource Inc, San Diego, CA, USA) following the manufacturer’s instructions.

### Statistical Analysis

Data were expressed as mean ± SEM. Statistical analysis was carried out with one-way ANOVA using SPSS 17.0 (SPSS Inc, Chicago, IL, USA). Statistical significance was set at *P* < 0.05.

## Results

### Curcumin Inhibits TGF-β2 Induced Proliferation of Mouse Lung Fibroblasts

**FIGURE 1:**
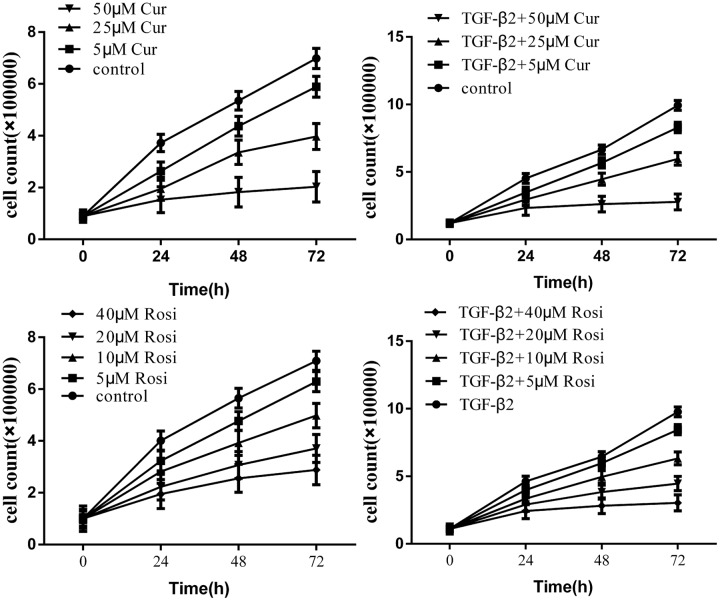
**Curcumin inhibits transforming growth factor β (TGF-β2) induced proliferation of mouse lung fibroblasts.** Mouse lung fibroblasts were treated with various concentrations of curcumin and rosiglitazone in the absence or presence of TGF-β2. Cell proliferation was assessed at 0, 24, 48, and 72 h. Data were presented as mean ± SEM. Cur, curcumin; Rosi, rosiglitazone.

Fibroblast proliferation plays a key role in the development of IPF. First, we assessed potential anti-proliferative effects of curcumin and rosiglitazone on fibroblasts. Mouse lung fibroblasts were treated with curcumin or rosiglitazone at various concentrations. The results showed that curcumin and rosiglitazone inhibited the proliferation of mouse lung fibroblasts in a dose and time dependent manner (**Figure 1**). The inhibition was significant both in the absence and in the presence of TGF-β2 (10 ng/ml).

### Curcumin Potently Inhibits TGF-β2 Induced Secretion of Collagen I

**FIGURE 2:**
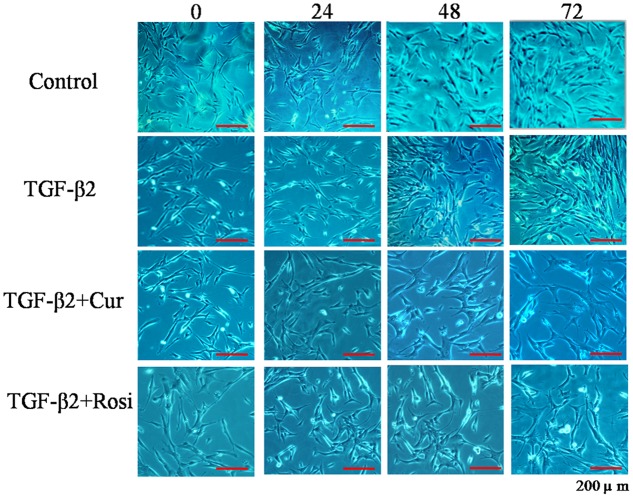
**Curcumin inhibits TGF-β2 induced morphological changes of mouse lung fibroblasts.** Mouse lung fibroblasts were treated with vehicle, TGF-β2, or TGF-β2 together with curcumin or rosiglitazone. Cell morphology was assessed at 0, 24, 48, and 72 h. Cur, curcumin; Rosi, rosiglitazone.

**FIGURE 3:**
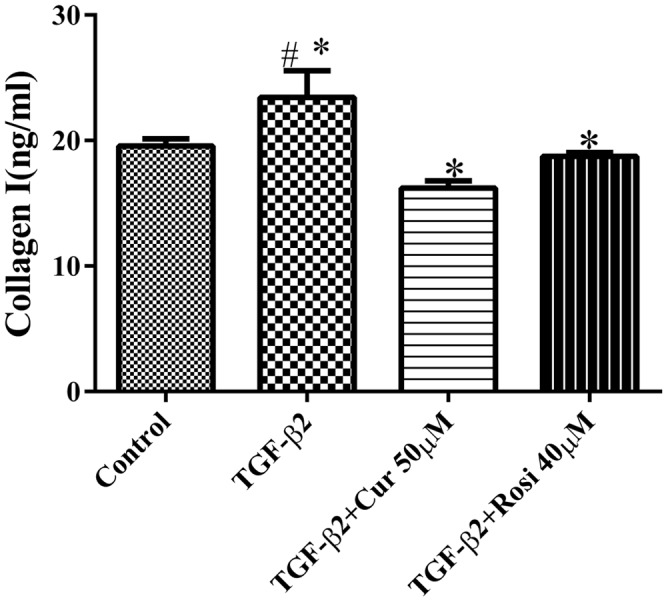
**Curcumin potently inhibits TGF-β2 induced secretion of collagen I.** Mouse lung fibroblasts were treated with 10 ng/ml TGF-β2 alone or after pretreatment with 50 μM curcumin or 40 μM rosiglitazone, and the secretion of collagen I was determined by ELISA. ^#^Significant increase after TGF-β2 treatment compared with control (*P* < 0.05, ANOVA).^∗^Significant reduction after curcumin or rosiglitazone treatment compared with TGF-β2 alone (*P* < 0.05, ANOVA). Cur, curcumin; Rosi, rosiglitazone.

TGF-β is known to increase collagen secretion by fibroblasts and promote their differentiation to myofibroblasts (Piersma et al., 2015). Next, we examined the effects of curcumin and rosiglitazone on TGF-β2 induced myofibroblast differentiation. Under microscope we found that TGF-β2 stimulated morphological change of mouse lung fibroblasts, but this could be partially reversed after treatment with curcumin or rosiglitazone (**Figure 2**). Furthermore, ELISA showed that TGF-β2 stimulated the secretion of collagen I, but this could be significantly inhibited by curcumin and rosiglitazone. In particular, curcumin was more effective at inhibiting collagen I secretion than Rosiglitazone (**Figure 3**). These data suggest that curcumin inhibits TGF-β2 induced myofibroblast differentiation.

### Curcumin Inhibits TGF-β2 Induced Myofibroblast Differentiation

**FIGURE 4:**
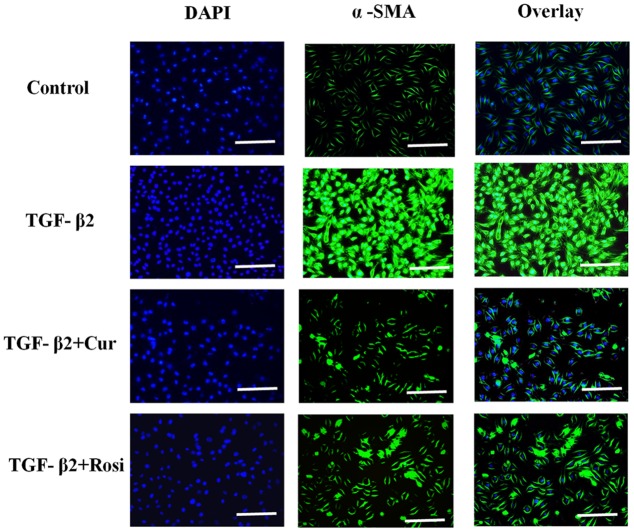
**Curcumin inhibits TGF-β2 induced α-SMA expression.** Mouse lung fibroblasts were pretreated with curcumin (50 μM) or rosiglitazone (40 μM) for 2 h and then treated with TGF-β2 (10 ng/ml) for 24 h. Immunocytochemical analysis for α-SMA (green, middle panel) was performed, DAPI (blue, left panel) was used to visualize the nuclei, and merged images were shown in right panel. Cur, curcumin; Rosi, rosiglitazone. Bar: 20 μm.

Expression of α-SMA is a key indicator of myofibroblast differentiation. To confirm that curcumin inhibits TGF-β2 induced myofibroblast differentiation, we performed immunocytochemical analysis of α-SMA expression in lung fibroblasts treated with curcumin (50 μM) or rosiglitazone (40 μM) in the presence of TGF-β2 (10 ng/ml). TGF-β2 treatment resulted in enhanced expression of α-SMA that was inhibited by curcumin and rosiglitazone. In particular, curcumin was more effective at inhibiting α-SMA expression than Rosiglitazone (**Figure 4**). These data demonstrate that curcumin inhibits TGF-β2 induced myofibroblast differentiation.

### Curcumin Upregulates PPAR-γ while Downregulates PDGFR-β during TGF-β2 Induced Myofibroblast Differentiation

**FIGURE 5:**
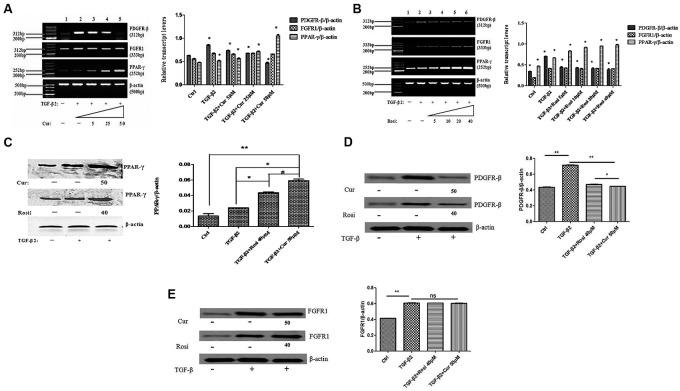
**Curcumin upregulates peroxisome proliferator-activated receptor gamma (PPAR-γ) and downregulates PDGFR-β during TGF-β2-induced myofibroblast differentiation.** **(A)** Mouse fibroblasts were treated for 24 h with TGF-β2 alone or together with 0–50 μM Cur. PPAR-γ, PDGFR-β, and FGFR1 mRNA levels were detected by PCR and normalized to β-actin mRNA. ^∗^*P* < 0.05, ANOVA. **(B)** Mouse fibroblasts were treated for 24 h with TGF-β2 alone or together with 0–40 μM Rosi. PPAR-γ, PDGFR-β, and FGFR1 mRNA levels were detected by PCR and normalized to β-actin mRNA. ^∗^*P* < 0.05, ANOVA. **(C)** Western blot analysis of PPAR-γ protein expression. ^∗^*P* < 0.05, ^∗∗^*P* < 0.05, ^#^*P* < 0.05, ANOVA. **(D)** Western blot analysis of PDGFR-β protein expression. ^∗^*P* < 0.05, ^∗∗^*P* < 0.05, ANOVA. **(E)** Western blot analysis of FGFR1 protein expression. ^∗∗^*P* < 0.05, ns *P* > 0.05, ANOVA. β-actin was loading control. Cur, Curcumin; Rosi, rosiglitazone.

Finally, we explored the mechanism by which curcumin inhibits TGF-β2 induced myofibroblast differentiation. Lung fibroblasts were treated with TGF-β2 alone or in combination with different concentrations of curcumin and rosiglitazone. PCR analysis showed that curcumin and rosiglitazone increased PPAR-γ mRNA expression but decreased PDGFR-β mRNA expression in TGF-β2-induced myofibroblasts in a dose-dependent manner. As the control, curcumin and rosiglitazone had no significant effect on the expression of FGFR1 mRNA (**Figures 5A,B**).

Furthermore, Western blot analysis demonstrated that curcumin and rosiglitazone significantly increased PPAR-γ expression at protein level (**Figure 5C**), decreased PDGFR-β expression at protein level (**Figure 5D**), but had no significant effect on FGFR1 protein expression (**Figure 5E**).

## Discussion

Idiopathic pulmonary fibrosis affects approximately five million people worldwide. IPF can lead to respiratory failure and death due to deteriorating respiratory function, and is the most common form of idiopathic interstitial pneumonias (IIPs), among which IPF has the worst prognosis with median survival of 3–5 years after diagnosis (Raghu et al., 2011; Olson and Swigris, 2012). The mechanism of IPF initiation and progression is poorly understood, and the disease is refractory to current therapies. Therefore, novel anti-fibrotic drugs are urgently needed for the treatment of IPF.

Curcumin has shown anti-fibrotic effects, but the underlying mechanisms remain poorly understood. In this study, we demonstrated that curcumin and rosiglitazone inhibited TGF-β2 induced alterations of biological characteristics of mouse lung fibroblasts, such as the suppression of proliferation (**Figure 1**) and differentiation (**Figure 2**) of mouse lung fibroblasts to myofibroblasts, the down-regulation of collagen secretion (**Figure 3**) and α-SMA expression (**Figure 4**). Notably, curcumin has stronger inhibitory effects on the proliferation and differentiation of mouse lung fibroblasts than rosiglitazone. Mechanistically, we found that the inhibitory effects of curcumin are associated with the upregulation of PPAR-γ and the downregulation of PDGFR-β (**Figure 5**).

Moreover, in this study we showed an antagonistic relationship between PPAR-γ and PDGF. Interestingly, a recent study reported that PPAR-γ inhibited hepatic stellate cells driven angiogenesis via the suppression of PDGF-β receptor expression and suggested that PPAR-γ could be a molecular target for preventing vascular remolding in hepatic fibrosis (Zhang F. et al., 2014). Therefore, we speculated that the activation of PPAR-γ results in the interruption of PDGF signaling pathway, leading to the inhibition of TGF-β2 driven differentiation of mouse lung fibroblasts to myofibroblasts. Further studies are needed to understand the interaction of PPAR-γ and PDGF in the pathogenesis of IPF.

This study has several limitations. We only used *in vitro* cell line and further *in vivo* studies in animal models are needed to confirm the anti-fibrotic effects of curcumin. While we linked anti-fibrotic effects of curcumin with the regulation of PPAR-γ and PDGF expression by curcumin, detailed underlying mechanisms remain elusive. There are several pathways associated with fibrotic processes during IPF. In this study, we only focused on TGF-β signaling pathway. Further studies are necessary to determine whether curcumin affects other signaling pathways that might be involved during fibroblast proliferation, differentiation into myofibroblasts and the expansion of ECM. Furthermore, TGF-β has been shown to induce the apoptosis of fibroblasts. It is worthy investigating the effects of curcumin on apoptotic pathways of fibroblasts.

## Conclusion

This study reveals novel mechanism by which curcumin inhibits TGF-β2 driven differentiation of mouse lung fibroblasts to myofibroblasts. Curcumin and PPAR-γ could potentially be used for effective treatment of IPF. Future *in vivo* studies are necessary to confirm the promising potential of curcumin as therapeutics for IPF.

## Author Contributions

DL conceived and designed the experiments. DL, LG, HZ, SP, and YW performed experiments. WZ and GH contributed reagents and materials. DL and HZ wrote the manuscript. All authors read and approved the final manuscript.

## Conflict of Interest Statement

The authors declare that the research was conducted in the absence of any commercial or financial relationships that could be construed as a potential conflict of interest.
